# Ghrelin Protects against Renal Damages Induced by Angiotensin-II via an Antioxidative Stress Mechanism in Mice

**DOI:** 10.1371/journal.pone.0094373

**Published:** 2014-04-18

**Authors:** Keiko Fujimura, Shu Wakino, Hitoshi Minakuchi, Kazuhiro Hasegawa, Koji Hosoya, Motoaki Komatsu, Yuka Kaneko, Keisuke Shinozuka, Naoki Washida, Takeshi Kanda, Hirobumi Tokuyama, Koichi Hayashi, Hiroshi Itoh

**Affiliations:** Department of Internal Medicine, School of Medicine, Keio University, Tokyo, Japan; Cardiological Center Monzino, Italy

## Abstract

We explored the renal protective effects by a gut peptide, Ghrelin. Daily peritoneal injection with Ghrelin ameliorated renal damages in continuously angiotensin II (AngII)-infused C57BL/6 mice as assessed by urinary excretion of protein and renal tubular markers. AngII-induced increase in reactive oxygen species (ROS) levels and senescent changes were attenuated by Ghrelin. Ghrelin also inhibited AngII-induced upregulations of transforming growth factor-β (TGF-β) and plasminogen activator inhibitor-1 (PAI-1), ameliorating renal fibrotic changes. These effects were accompanied by concomitant increase in mitochondria uncoupling protein, UCP2 as well as in a key regulator of mitochondria biosynthesis, PGC1α. In renal proximal cell line, HK-2 cells, Ghrelin reduced mitochondria membrane potential and mitochondria-derived ROS. The transfection of UCP2 siRNA abolished the decrease in mitochondria-derived ROS by Ghrelin. Ghrelin ameliorated AngII-induced renal tubular cell senescent changes and AngII-induced TGF-β and PAI-1 expressions. Finally, Ghrelin receptor, growth hormone secretagogue receptor (GHSR)-null mice exhibited an increase in tubular damages, renal ROS levels, renal senescent changes and fibrosis complicated with renal dysfunction. GHSR-null mice harbored elongated mitochondria in the proximal tubules. In conclusion, Ghrelin suppressed AngII-induced renal damages through its UCP2 dependent anti-oxidative stress effect and mitochondria maintenance. Ghrelin/GHSR pathway played an important role in the maintenance of ROS levels in the kidney.

## Introduction

Ghrelin is a 28-amino acid peptide containing an octanoyl modification isolated from the stomach and recognized as an endogenous ligand for the growth hormone (GH) secretagogue receptor (GHSR) [1], [2]. In addition to its GH-releasing effects, various activities have been described so far that are consistent with the wide tissue distribution of GHSR including the kidney. A recent study demonstrated that the exogenous injection of Ghrelin protected against acute kidney injuries [3], [4].

In chronic kidney damages, several humoral and hemodynamic factors have been shown to accelerate progression, including the activation of renin-angiotensin systems (RAS) [5]. AngII is the effector of RAS-induced renal tissue damage through multiple mechanisms, including pro-inflammatory or pro-fibrotic effects [6]. AngII has also been reported to provoke oxidative stress, mainly through the activation of NADPH-oxidase and accelerated tissue senescent changes [7], [8]. This type of premature senescent reaction has been called stress-induced senescence, and it is accepted that premature renal senescence causes the deterioration of renal function and accelerates the progression of chronic kidney disease [9], [10]. In diabetic nephropathy, renal tissues and tubular cells are in a senescent state in comparison to the normal kidney [11], [12], which is demonstrated by increased activity of β-galactosidase when assayed at pH 6. This type of β-galactosidase is known as senescence-associated (SA) β-gal, and it is often used as a marker for a senescent cell [13]. It has also been demonstrated that senescent cells express and produce various kinds of growth factors or cytokines, such as transforming growth factor-β (TGF-β) or plasminogen activator inhibitor-1 (PAI-1), which enhances tissue fibrosis and contributes to the functional decline [12].

Recent studies revealed that Ghrelin exerts its anti-oxidative function by reducing the production of reactive oxygen species (ROS) from mitochondria [14]. Ghrelin has been demonstrated to increase the expression of mitochondrial uncoupling protein UCP2 in neuronal cells [15], diminishing the mitochondrial membrane potential, reducing excessive oxidative phosphorylation reactions in mitochondria, and downregulating mitochondria ROS production [14]. Given the expression of Ghrelin and its receptor GHSR in the kidney [16], it is surmised that Ghrelin protects against the progression of chronic kidney damage and renal premature senescence.

In this study, the protective effects of Ghrelin on chronic renal damages induced by AngII infusion were investigated. We further examined the role of endogenous Ghrelin/GHSR system in the kidney by using GHSR-null mice.

## Results

### Ghrelin attenuates renal tubular damages in AngII-infused mice models

**Figure 1:**
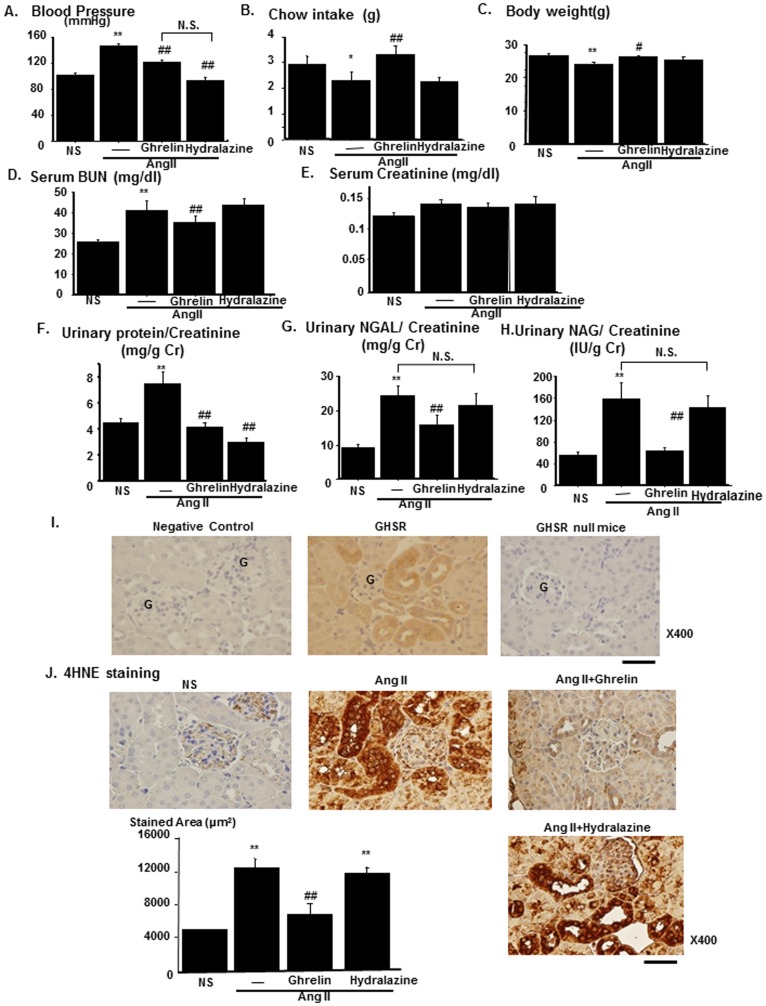
Amelioration of renal tubular damages and increased renal oxidative stress by Ghrelin in AngII-infused mice. The effects of Ghrelin treatment on the phenotypes of AngII-infused mice. Blood pressures (A), daily chow intake (B) and body weight (C) were compared among saline-infused mice (NS), AngII-infused mice (AngII), and AngII-infused mice treated with Ghrelin (AngII+Ghrelin) or Hydralazine (AngII+Hydralazine). Serum levels of blood urea nitrogen (BUN, D) and creatinine (E), urinary excretion of protein (F), neutrophil gelatinase-associated lipocalin (NGAL, G), n-acetyl-galactasaminase (NAG, H) were compared among the experimental groups. (I) Representative immunostaining for the Ghrelin receptor (Growth hormone secretagogue receptor, GHSR) is shown in the middle panel. Negative control without using anti-GHSR antibody is shown in the left panel. The staining of GHSR in the kidney of GHSR null mice is also shown in the right panel. Scale bar, 50 µm. G represents glomerulus. (J) Representative immunostaining for 4-Hydroxynonenal-2-nonenal (4HNE) of four experimental groups. Bar graphs represent the quantification of immunostained areas. Scale bar; 50 µm. **p<0.01 vs. NS, *p<0.05 vs. NS, ##p<0.01 vs. AngII, #p<0.05 vs. AngII, N.S. represents no significant difference. n = 8.

Ghrelin treatment lowered the blood pressure of AngII-infused mice (AngII) as compared to that in saline-infused mice. To rule out the effects on blood pressure, the anti-hypertensive drug, hydralazine was given orally to AngII-infused mice. The treatment with hydralazine at the dose of 25 mg/kg/day [17] lowered blood pressure to levels similar to those with Ghrelin treatment (Figure 1A). Chow intake was decreased by AngII and this decrease was reversed by Ghrelin through its orexigenic effects (Figure 1B). Body weight was decreased in AngII-infused mice and this decrease was attenuated by Ghrelin (Figure 1C). Serum blood urea nitrogen (BUN) levels were increased in AngII-infused mice and this increase was attenuated by Ghrelin but not by hydralazine, although serum creatinine levels were not altered. (Figure 1D and 1E). Urinary protein excretion was increased in AngII-infused mice and this increase was attenuated both by Ghrelin and by hydralazine, indicating that the inhibitory effects by Ghrelin on urinary protein excretion were by blood pressure-dependent mechanism (Figure 1F). Urinary excretion of both neutrophil galatinase-associated lipocalin (NGAL) and n-acetyl-galactasaminase (NAG), markers for proximal tubular damages, increased in AngII-infused mice, and these increases were attenuated in Ghrelin-treated mice. However, hydralazine had no effects on urinary excretion of either NGAL or NAG (Figure 1G and 1H). In immunohistochemistry using antibody against GHSR, GHSR was expressed in the renal tubular area but not in the glomerular area (Figure 1I). Renal tissue oxidative stress levels were increased in the proximal tubular area in AngII-infused mice as assessed by the immunostaining for 4-Hydroxynonenal-2-nonenal (4HNE). This increase was abolished by Ghrelin treatment, although hydralazine had no effect (Figure 1J).

### Ghrelin ameliorates renal senescence changes and fibrosis induced by AngII

**Figure 2:**
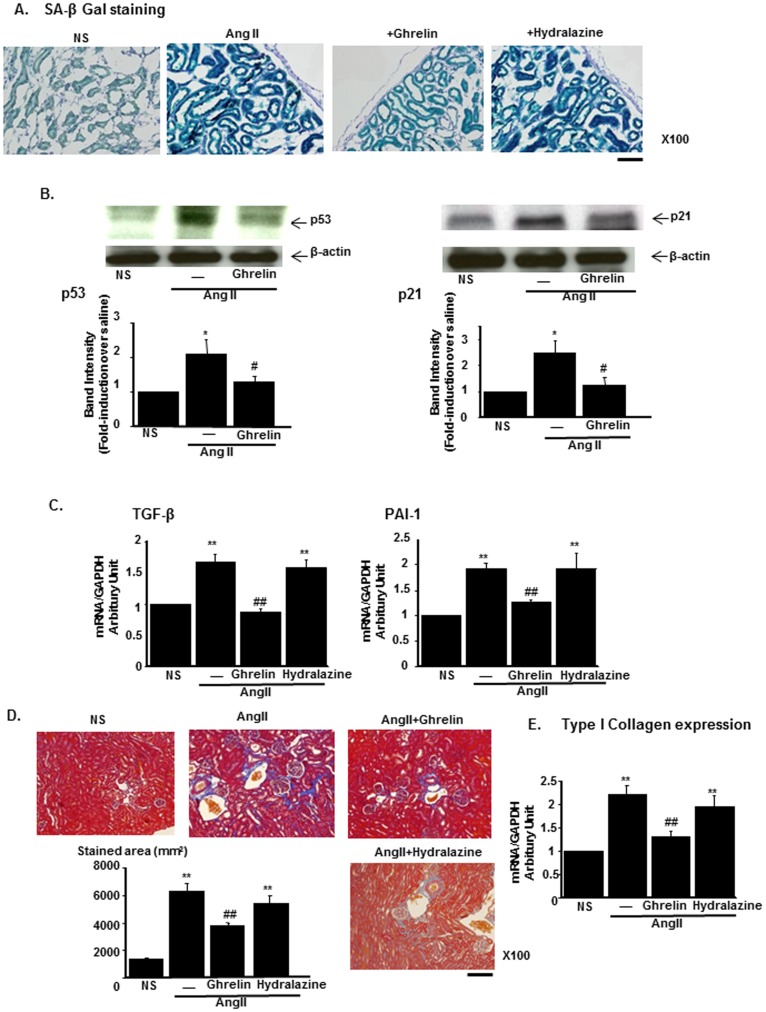
Amelioration of renal tissue senescent and fibrotic changes by Ghrelin in AngII-infused mice. (A) Representative staining of senescence-associated β-Galactosidase (SA β-Gal). Scale bar, 100 µm. NS represents normal saline. (B) The protein expressions of p53 (left) and p21 (right) in saline-infused mice (NS), AngII-infused mice (AngII), and AngII-infused mice treated with Ghrelin. The representative immunoblotting (upper panel) and the results of densitometry analysis (lower panel) are shown. (C) The expression of TGF-β (left) and PAI-1 (right) mRNA in mice of each group. (D) The representative results of Masson-Trichrome staining of each experimental group. Bar graphs represent the quantification of fibrotic areas. Scale bar; 100 µm. (E) The mRNA expression levels of type I collagen in the kidney of each group. **p<0.01 vs. NS, *p<0.05 vs. NS, ##p<0.01 vs. AngII, #p<0.05 vs. AngII, n = 8.

Oxidative stress has been reported to induce cellular or tissue senescence, which is known as stress-induced premature senescence [9]. Anti-oxidative stress effects by Ghrelin in the kidney are suggested to cause renal anti-senescence effects, as previously reported in other tissues [18]. After AngII infusion, SA β-Gal staining revealed that staining levels were increased, particularly in tubular cells. This increase was attenuated by Ghrelin (Figure 2A). The cell cycle negative regulator p53 and its downstream target gene p21 were among the markers for cellular senescence. AngII infusion significantly increased the expression of both p53 and p21. These inductions were blocked by the treatment with Ghrelin (Figure 2B). The changes in the inflammatory cytokines characteristics of senescence reaction, including those in TGF-β and PAI-1, were examined. AngII infusion to mice increased the expression of both TGF-β and PAI-1 in the kidney. These increases were significantly blocked by Ghrelin (Figure 2C, left and right panel, respectively). These cytokines contribute to the renal fibrotic changes [19] and we evaluated interstitial fibrosis by Masson-trichrome staining. AngII infusion increased perivascular fibrosis and this change was attenuated by the treatment with Ghrelin but not by the treatment with hydralazine (Figure 2D). Consistently, the expression levels of type I collagen in the kidney were increased in AngII-infused mice and this increase was attenuated by Ghrelin treatment (Figure 2E).

### Ghrelin increased UCP2 expression and the number of mitochondria in the kidney

**Figure 3:**
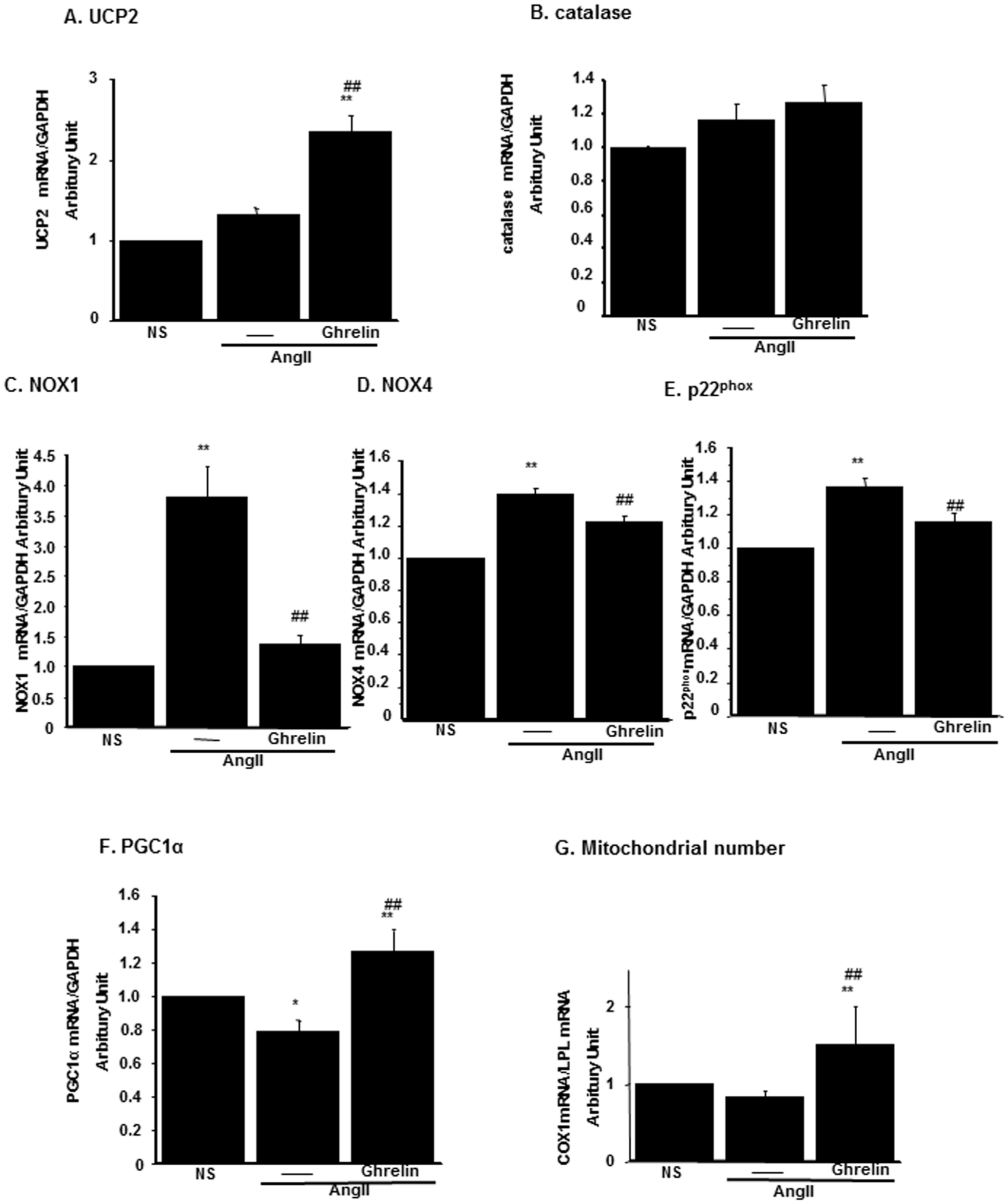
The mRNA expressions of anti-oxidative stress molecules and mitochondria number in the kidney. The mRNA expressions of UCP2 (A), catalase (B), NADPH oxidases, NOX1 (C), NOX4 (D), p22phox (E) and PGC1α (F) in saline-infused mice (NS), AngII-infused mice (AngII), and AngII-infused mice treated with Ghrelin were examined by real-time PCR. (G) The mitochondria number was assessed as real-time PCR for mitochondria-specific molecule, COX1 as described in Materials and Methods. **p<0.01 vs. NS, *p<0.05 vs. NS, ##p<0.01 vs. AngII, #p<0.05 vs. AngII, n = 8.

We examined the expression of various molecules related to oxidative stress. The expression levels of UCP2 were increased in Ghrelin-treated mice although AngII infusion failed to induce UCP2 expression (Figure 3A). The expression of catalase was not altered either by AngII or by Ghrelin (Figure 3B). The expressions of NADPH oxidase (NOX) contributing to the ROS production by AngII were examined and the expressions of two isoforms of NOX1 and NOX4 were increased by AngII (Figure 3C and 3D, respectively). The expression of the NOX subunit p22phox was also increased by AngII (Figure 3E). These molecules were downregulated by Ghrelin. The expression levels of peroxisome proliferator-activated receptor-γ coactivator 1α (PGC-1α), a key regulator of mitochondria number, increased by treatment with Ghrelin (Figure 3F). The number of mitochondria also increased in Ghrelin-treated mice (Figure 3G). These data suggested that Ghrelin not only attenuated AngII-induced ROS production, but also increased the number of mitochondria in the kidney through both inhibitory effect on AngII-dependent ROS production and its own anti-oxidative stress effects.

### Ghrelin reduced mitochondria ROS through UCP2 dependent mechanism

**Figure 4:**
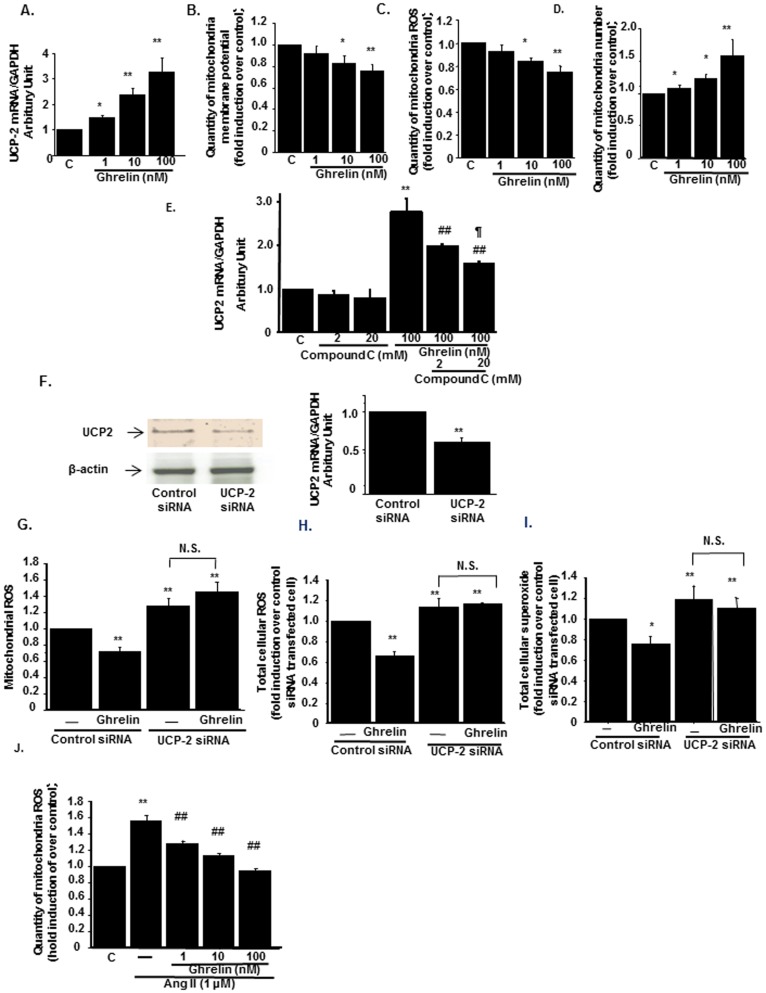
Mitochondria-derived ROS was reduced by Ghrelin through the induction of UCP2. (A) The effects of Ghrelin on mitochondria-derived UCP2 mRNA levels. (B) Mitochondrial membrane potential was measured by the specific dye as described in Materials and Methods. **p<0.01 vs. control cells, n = 8. (C, D) The effects of Ghrelin on mitochondria-derived ROS levels (C) and mitochondria number (D) in HK-2 cells. **p<0.01 vs. control HK-2 cells, *p<0.05 vs. control, n = 8. (E) The effects of AMP-kinase inhibitor on Ghrelin-induced UCP2 upregulation. Compound C, AMP-kinase inhibitor at the concentrations of 2 and 20 µM was pretreated 30 minutes before the Ghrelin administration to HK-2 cells. **p<0.01 vs. control HK-2 cells, ##p<0.01 vs. HK-2 cells treated with 100 nM of Ghrelin, ¶p<0.05 vs. Ghrelin-treated cell with 2 µM of Compound C administration, n = 8. (F) Knock-down of UCP2 protein and mRNA were shown in the representative immunoblotting (left panel) and real-time PCR (right panel), respectively. **p<0.01 vs. control siRNA-transfected cells, n = 6. (G–I) Mitochondria-derived ROS (G), total cellular ROS (H), and total cellular superoxide (I) were measured after the transfection of UCP2 siRNA or control siRNA. HK-2 cells were transfected with siRNA and treated with or without 100 nM of Ghrelin **p<0.01 vs. control siRNA-transfected cells without Ghrelin. N.S. represents no significant difference. n = 8. (J) The effects of Ghrelin on AngII-induced Mitochondrial ROS production. HK-2 cells were treated with 1 nM, 10 nM, and 100 nM of Ghrelin 30 minutes before the treatment with 1 mM of AngII. respectively. **p<0.01 vs. control cells, *p<0.05 vs. control cells, ##p<0.01 vs. AngII-treated HK-2 cells, #p<0.05 vs. AngII-treated HK-2 cells, n = 8.

By utilizing HK-2 cells, a human renal proximal tubular cell line, we further examined anti-oxidative stress effects of Ghrelin. HK-2 cells expressed both the mRNA and protein of GHSR (Figure S1A). Ghrelin increased the expression of UCP2 in a dose-dependent manner (Figure 4A). UCP2 reduces mitochondrial membrane potential and inhibits the excessive ATP production and mitochondrial ROS production [20]. Treatment with Ghrelin reduced membrane potential in a dose-dependent manner (Figure 4B). Due to the reduction of membrane potential, Ghrelin decreased mitochondria-derived ROS levels (Figure 4C). In addition, treatment with Ghrelin significantly increased the mitochondria number in a dose-dependent manner (Figure 4D). Ghrelin has been shown to activate AMP-kinase in central nervous systems [21]. The upregulation of UCP2 by Ghrelin was blocked by AMP-kinase inhibitor, Compound C in a dose-dependent manner (Figure 4E). These results implied that UCP2 was upregulated by Ghrelin through AMP kinase activation. We next designed siRNA for UCP2, which successfully knockdown both protein and mRNA expressions of UCP2 (Figure 4F, left and right panels, respectively). The expressions of other genes related to antioxidative stress or other isoforms of UCP were not affected by this siRNA, which confirmed the specificity of this siRNA (Figure S1B–S1E). Transfection of siRNA for UCP2 abolished the reduction of mitochondria-derived ROS production by Ghrelin (**Figure 4G**). UCP2 gene knockdown also abrogated the effects of Ghrelin on the production of ROS and superoxide from total cellular origin (Figure 4H and 4I, respectively). Ghrelin also reduced AngII-induced mitochondria-derived ROS production (Figure 4J).

### Ghrelin attenuates AngII-induced renal proximal tubular cell senescence

**Figure 5:**
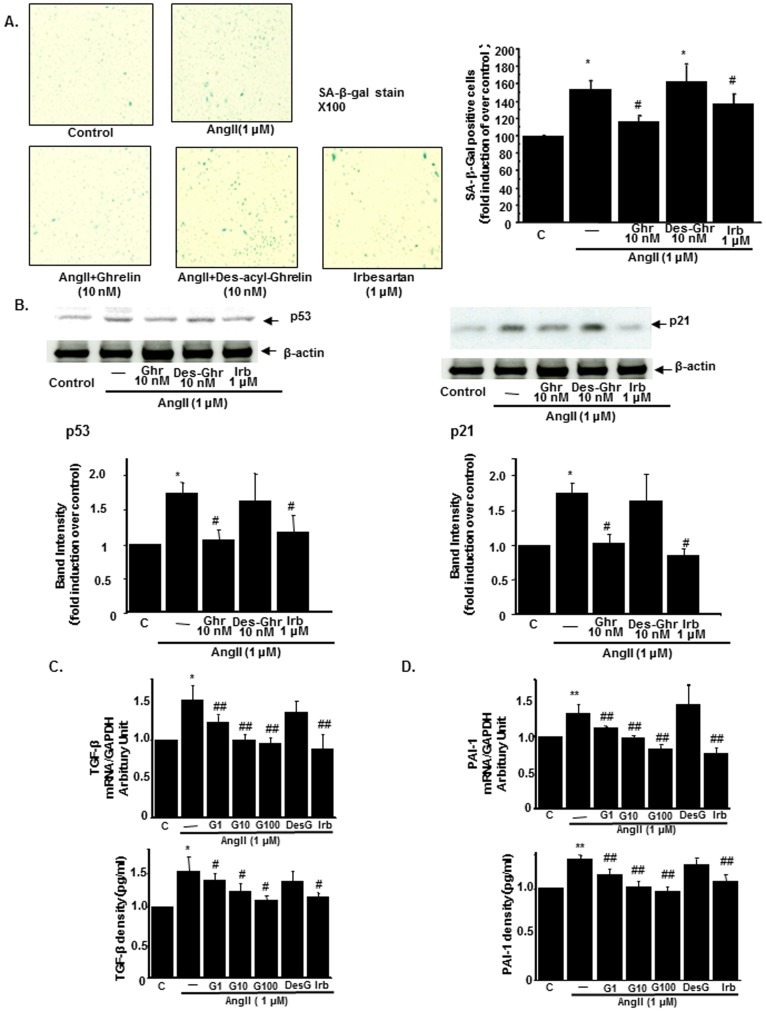
The amelioration of cellular senescent changes in AngII-treated HK-2 by Ghrelin. (A) Representative staining of senescence-associated β-Galactosidase (SA β-Gal) in untreated HK-2 cells (control), AngII-treated HK-2 cells (AngII, 1 µM), and AngII-treated with the pretreatment of 10 nM Ghrelin (AngII+Ghrelin), 10 nM Des-acyl-Ghrelin (AngII+Des-acy-Ghrelin), or 1 µM AngII type 1 receptor antagonist, irbesartan (left panel). Bar graphs represent the quantification of stained cells (right panel). (B) The protein expressions of p53 (left) and p21 (right) in HK-2 cells. The representative immunoblotting (upper panel) and the results of densitometry analysis (lower panel) were shown. (C) The expression of TGF-β mRNA in HK-2 cells (upper panel) and the concentration of TGF-β in the medium of HK-2 cells (lower panel). (D) The expression of PAI-1 mRNA in HK-2 cells (upper panel) and the concentration of PAI-1 in the medium of HK-2 cells (lower panel). C; control cells, AngII; HK-2 cells treated with 1 µM of AngII, G1, G10, G100; HK-2 cells treated with 1 nM, 10 nM, and 100 nM of Ghrelin, respectively, Des-G; HK-2 cells treated with 10 nM of Des-acyl-Ghrelin, Irb; HK-2 cells treated with 1 µM of irbesartan **p<0.01 vs. control HK-2 cells, *p<0.05 vs. control HK-2 cells, ##p<0.01 vs. AngII-treated HK-2 cells, #p<0.05 vs. AngII-treated HK-2 cells, n = 8.

The anti-senescence effects of Ghrelin in renal tubular cells were also investigated by using HK-2 cells. With AngII treatment, the population of senescent cells stained in the SA-β Gal assay was increased. This increase was inhibited by pretreatment with Ghrelin as well as with AngII receptor blockade, irbesartan but not with Des-acyl Ghrelin, the inactive form of Ghrelin (Figure 5A). AngII also increased the expressions of p53 and p21 in HK-2 cells. These increases were downregulated by the pretreatment with Ghrelin but not by Des-acyl Ghrelin (Figure 5B). The mRNA expression of TGF-β and the secretion of TGF-β in the medium were increased by AngII. These effects of AngII were inhibited by the pretreatment with Ghrelin (Figure 5C). Similar results were obtained for the expression and secretion of PAI-1 in HK-2 cells (Figure 5D).

### Renal tubular damages were evident in GHSR-null mice

**Figure 6:**
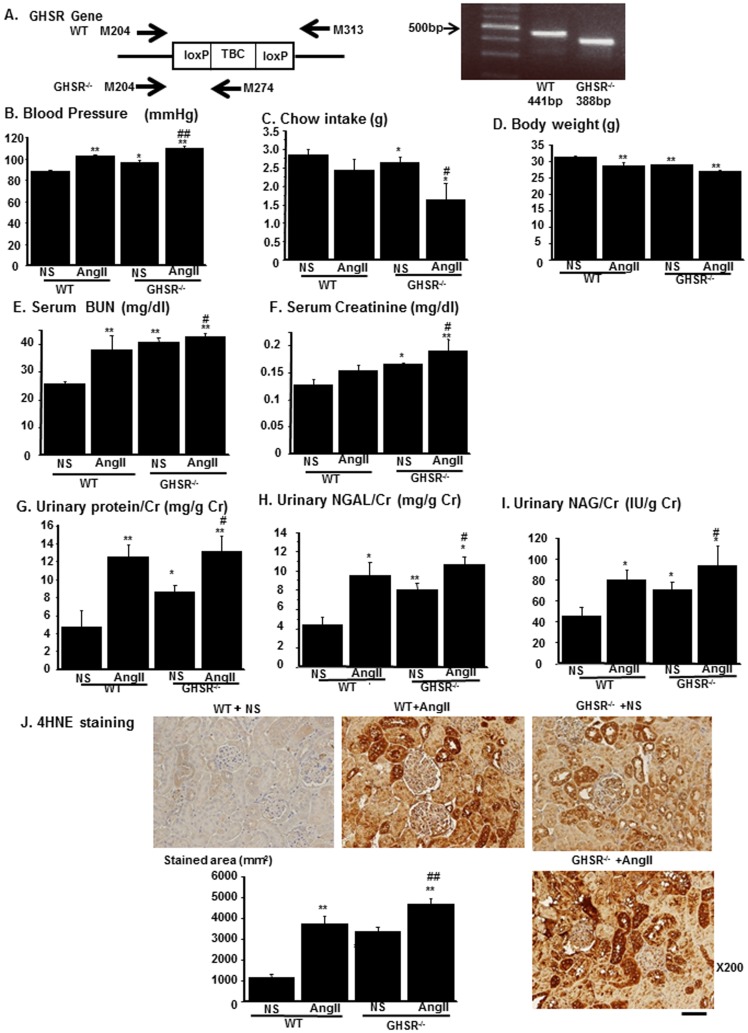
Phenotype of GHSR-null mice. (A) The primers used for the genotyping in the PCR (left) and representative results of genotyping (right). The primers used are indicated as arrows. TBC, transcription blocking cassette. (B–J) The phenotype differences among the four experimental groups: WT or GHSR^−/−^ mice infused with normal saline (NS) or AngII. Blood pressure (B), daily chow intake (C), body weight (D) were compared among the four groups. Serum levels of blood urea nitrogen (BUN, E) and creatinine (F) and urinary excretion of protein (G), neutrophil gelatinase-associated lipocalin (NGAL, H), and n-acetyl-galactasaminase (NAG, I) were compared among the four experimental groups. Urinary excretion of each marker was normalized by that of creatinine. (J) Representative immunostaining for 4-Hydroxynonenal-2-nonenal (4HNE) of four experimental groups. Bar graph represents the quantification of immunostained areas. Scale bar; 50 µm. **p<0.01 vs. WT+NS, *p<0.05 vs. WT+NS, ##p<0.01 vs. GHSR^−/−^+NS, #p<0.05 vs. GHSR^−/−^+NS, n = 8.

We further delineated the role of endogenous Ghrelin in the kidney by using GHSR-null mice [22]. Real-time PCR revealed that GHSR-null mice lacked expression of GHSR (Figure 6A). GHSR-null mice (KO) and wild-type (WT) littermates were infused with AngII or normal saline (NS). We compared various phenotype among the four group, WT mice infused with NS (WT+NS) or AngII (500 ng/kg/day, WT+AngII) and KO mice infused with NS (KO+NS) or AngII (KO+AngII). Systolic blood pressure was increased in KO+NS as compared with that in WT+NS and AngII further increased systolic blood pressure in KO mice (KO+NS vs. KO+AngII) (Figure 6C). Daily chow intake was reduced in KO mice and body weight was decreased in KO+NS as compared with those in WT. These changes were augmented by AngII infusion (Figure 6D and 6E, respectively). Serum BUN and creatinine levels were increased in KO+NS as compared to those in WT+NS, which were further aggravated by AngII infusion (Figure 6F and 6G, respectively). Urinary protein excretion (Figure 6H) as well as urinary excretion of renal tubular markers, NGAL (Figure 6I) and NAG (Figure 6J), were increased in KO+NS as compared with those in WT+NS. These increases were augmented by AngII infusion (KO+NS vs. KO+AngII). 4HNE staining showed that ROS levels in the kidney increased in KO+NS as compared with those in WT+NS. These increases were enhanced by AngII infusion (KO+NS vs. KO+AngII) (Figure 6K). These data demonstrated that endogenous Ghrelin was involved in the regulation of renal ROS levels in the kidney and had a protective role against renal damages by ROS. We further treated AngII-infused GHSR-null mice with Ghrelin by daily intraperitoneal injection for 14 days. The treatment of Ghrelin failed to attenuate the AngII-induced increase in blood pressure, serum BUN concentration, serum creatinine concentration, urinary protein excretion, and urinary excretion of NAG and NGAL (Figure S2B–S2G, respectively). Similarly, Ghrelin had no effects on the expression of UCP2 and on AngII-induced oxidative stress increase in AngII-infused GHSR-null mice (Figure S2H and S2I, respectively). These data indicated that the effects of Ghrelin against AngII-induced renal damages were mediated by GHSR dependent mechanism.

### GHSR-null mice exhibited renal senescence, fibrosis and mitochondrial loss

**Figure 7:**
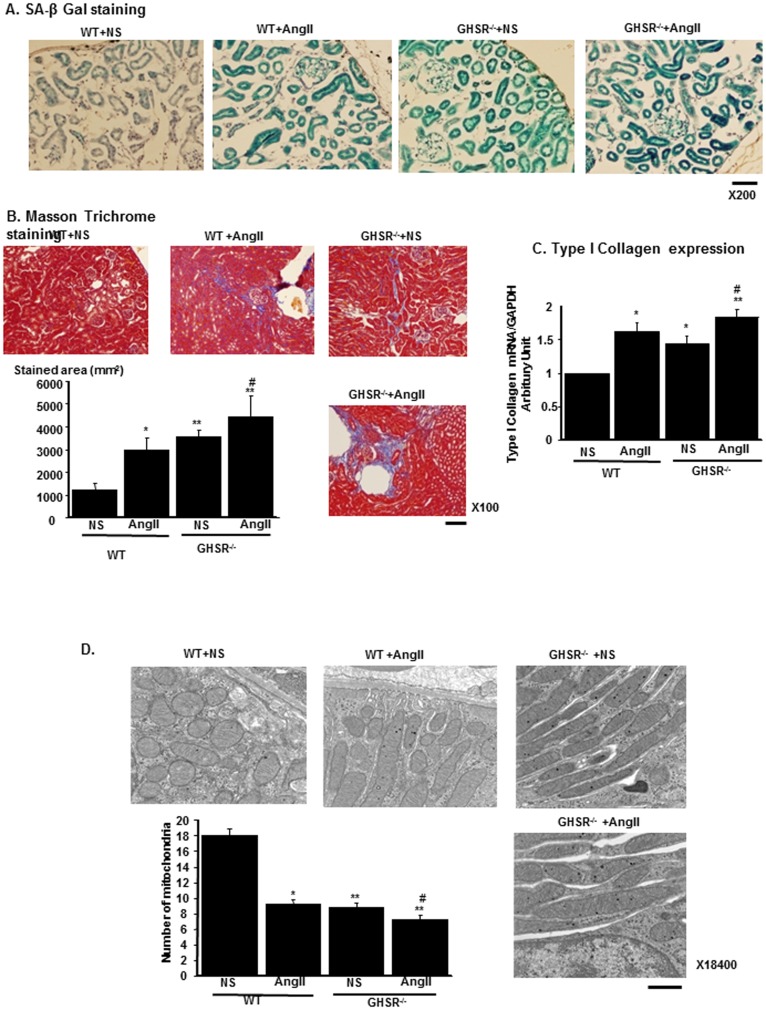
Tissue senescent, fibrotic changes and electromicroscopic findings in GHSR-null mice. (A) Representative staining of senescence-associated β-Galactosidase (SA β-Gal) in WT wild type mice (WT) or GHSR-null mice (GHSR^−/−^) infused with normal saline (NS) or AngII. Scale bar, 50 µm. (B) The representative results of Masson-Trichrome staining of each experimental group. Bar graphs represent the quantification of fibrotic areas. Scale bar, 100 µm. (C) The mRNA expression levels of type I collagen of each experimental group. (D) Electron microscopic findings of mitochondria show that the number of mitochondria was reduced and that morphology of mitochondria was altered in GHSR-null mice (GHSR^−/−^) with NS in comparison to that in WT+NS. Bar graph represents the result of number of mitochondria in the field of electron microscope in each mice group. Scale bar; 1 µm. **p<0.01 vs. WT+NS, *p<0.05 vs. WT+NS, #p<0.01 vs. GHSR^−/−^+NS, n = 6.

The increase in ROS levels in KO mice suggest that enhanced senescence changes and fibrotic changes in the kidney are evident in KO mice. SA-β Gal staining revealed that staining intensity increased in KO+NS as compared with that in WT+NS. AngII also increased SA-β Gal staining both in WT and in KO (Figure 7A). Masson-Tichrome staining revealed that fibrotic changes increased in KO+NS as compared with in WT+NS. AngII also enhanced fibrotic changes both in WT and in KO (Figure 7B). Similar results were obtained as regards the renal expression levels of type I collagen, the molecular marker for tissue fibrosis (Figure 7C). Our *in vitro* findings demonstrate that these changes were caused by the mitochondrial damages and increased mitochondrial ROS levels. In the electromicroscopic finding, the number of mitochondria was reduced in KO+NS as compared to that in WT+NS. This reduction of mitochondria number was enhanced by AngII infusion. In mitochondrial morphology, mitochondria were elongated in KO+NS, and this morphological alteration was enhanced in KO+AngII (Figure 7D).

## Discussion

**Figure 8:**
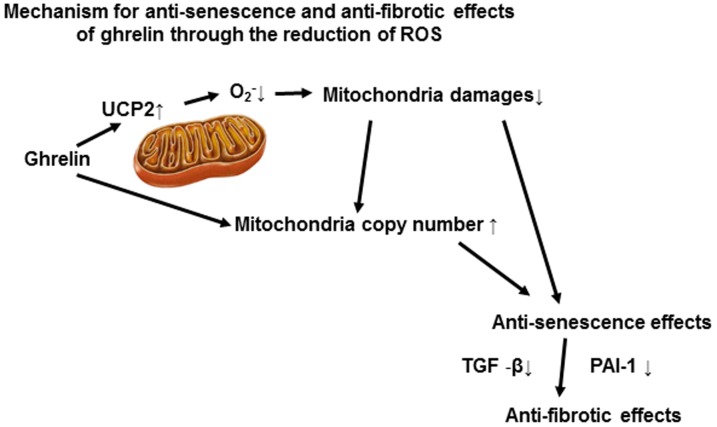
Schema depicting the renal protective effects by Ghrelin. Ghrelin upregulated UCP2 and decreased mitochondria-derived oxidative stress levels. These effects mitigated mitochondria damages and retained the mitochondria number, contributory to its anti-senescent effects. Anti-senescent effects by ghrelin was related to the downregulation of TGF-β and PAI-1, pro-fibrotic genes and inhibited the tissue fibrotic changes.

We demonstrated that the gut peptide Ghrelin exerts a renal protective effect by reducing oxidative stress levels and by retaining mitochondria, through the induction of mitochondria UCP2 expression and PGC1α expression. These effects contributed to the ameliorations of tissue senescent changes and fibrosis in AngII-induced renal damages (Figure 8). We also demonstrated that anti-oxidative effect by endogenous Ghrelin had also an important role in maintenance of redox state in the kidney.

UCP2 maintains the membrane potential and regulates mitochondrial ROS production during oxidative phosphorylation [20]. It has also been demonstrated that superoxide activates UCP2, and this regulatory mechanism functions as a feedback mechanism for excess oxidative stress [23]. In central nervous system [24] and human vascular smooth muscle cell [25], AngII-induced oxidative stress was silenced by the upregulation of UCP2 through the activation of p38 MAP kinase [24]. In our AngII-infusion kidney and also in AngII-stimulated HK-2 cells, the upregulation of UCP2 was not observed (Figure 3A) and this deficiency of the compensatory mechanism in renal tubular cells might aggravate tissue damages by oxidative stress by AngII. However, this aggravation was ameliorated by Ghrelin-induced UCP2 upregulation by its action to mitochondria. In human vascular smooth muscle cells, adenovirus-mediated gene transfer of UCP2 ameliorated AngII-induced ROS production [25], which was consistent with our results in renal tubular cells. Previous reports demonstrated that UCP2 was upregulated by the AMP-kinase pathway [26]. AMP-kinase activation also resulted in the increased mitochondria number through the activation of PGC1α [27], [28]. Ghrelin has been reported to activate AMP-kinase pathway in appetite regulation through the GHSR-dependent pathway [21]. The effects by Ghrelin on AMP-kinase activation would contribute to the UCP2 upregulation as shown in Figure 4E and also to the increase in the mitochondria number.

The production of ROS by AngII are main signal intermediates involved in renal pathophysiology [29], [30]. AngII-induced ROS are important for renal inflammation and fibrosis [31]. In our AngII-infused model, AngII increased the expression of NOX1 and NOX4, major isoforms of NOX in the kidney. It has been found that AngII stimulates upregulation of various NADPH oxidase subunits, including NOX1, p47phox, p67phox, and p22phox in cytosolic fraction [29]. Ghrelin decreased the expression levels of NOX1 and p22phox. NOX expressions have been implicated to be regulated by the redox state [32], [33]. Therefore, it can be surmised that Ghrelin partially downregulated AngII-induced NOXs increase through the potent reduction in oxidative stress [32], [33] (Figure 3C–3E). However, these cytosolic ROS production was not completely ameliorated, which results were consistent with the results that AngII increased renal tissue oxidative stress levels and tissue damages even in the GHSR-null mice. A recent study also suggested AngII-stimulated mitochondrial ROS generation through the opening of mitochondrial K_ATP_ channels [34] and induced expression of mitochondrial NOX4 in renal tubular cells [35]. Our group previously demonstrated that AngII increased mitochondria-derived ROS and the number of mitochondria in muscle tissues [36]. The present data revealed that treatment with Ghrelin downregulated the increase in NOX4 expression in AngII-infused mice kidney. Therefore, Ghrelin reduced AngII-induced ROS production by mitochondria-dependent and/or independent mechanisms.

Mitochondria are sensitive to oxidative stress because its DNA lacks the histone protein and easily breaks down by ROS. Kidney proximal tubular cells possess large numbers of mitochondria and are highly dependent on mitochondrial energy production for their proper function [37]. Therefore, the damages of proximal tubular cells were dependent on the mitochondria damages or mitochondria loss evoked by tissue oxidative stress. Especially in acute kidney injuries such as ischemia-reperfusion injuries [38] or cisplatin-induced kidney damages [39], mitochondria damages in proximal tubules contributed to the decline in renal function. We previously reported that the overexpression of NAD-dependent deacetylase Sirt1 in the proximal tubules reversed the decline in renal function by cisplatin-induced and ischemia-reperfusion renal damages by reducing oxidative stress and retaining mitochondria number [40]. In our AngII-infused models, the decreased number of mitochondria was induced presumably by the increased ROS production in the proximal renal tubules and renal proximal tubular damages were prominent as evaluated by urinary excretion of proximal tubular cell markers. Ghrelin treatment reduced mitochondria-derived ROS production, induced PGC1α and restored mitochondria number in the kidney of these mice. These effects by Ghrelin ameliorated renal tubular damages by AngII.

In the present study, we described the phenotype of GHSR-null mice. GHSR null mice already exhibited renal tubular damages, renal dysfunction and increase in oxidative stress levels. We also observed the increase in the number of elongated mitochondria and the decrease in total mitochondria number in proximal tubular cells in GHSR null mice. The elongated mitochondria indicated the accumulation of oxidative stress in mitochondria [41]. In addition, the elongated mitochondria develop due to the escape from the autophagy process [41]. Therefore, it is concluded that the damaged mitochondria were accumulated in the proximal tubules of GHSR-null mice. Moreover, GHSR-null mice exhibited the decline in renal function as indicated by increase in plasma creatinine levels. Our data demonstrated that Ghrelin/GHSR pathway stabilizes the ROS status and mitochondria quality and maintains renal function by the regulation of mitochondria oxidative stress levels.

In previous reports, Ghrelin mitigated acute renal damages induced by ischemia and reperfusion injuries [3]. These effects were mediated through the activation of the growth hormone-insulin-like growth factor 1 pathway. In another study, Ghrelin treatment protected against acute endotoxemia-induced kidney injury through the inhibition of multiple proinflammatory cytokines [4]. These studies did not demonstrated direct effects of Ghrelin on the kidney. Our study provides evidence for novel direct protective effects of Ghrelin on the tubular cells through anti-oxidative effects. The mRNA expressions of GHSR have been confirmed in the kidney, according to a recent study [16]. In the present study, immunostaining for GHSR was detected in the tubular area including in the proximal portion of tubules (Figure 1I). In addition, HK-2 cells of the proximal tubular cell line expressed both the mRNA and protein of GHSR (Figure 4A). The treatment with Ghrelin ameliorated the damages to proximal tubules as assessed by the proximal tubular marker, NAG and NGAL (Figure 1G and 1H). Our data indicates the possibility that GHSR is expressed in the proximal portion, and that Ghrelin exerts its renal protective effect directly through GHSR in the kidney.

In conclusion, we demonstrated that Ghrelin ameliorated AngII-induced renal damages through the reduction of oxidative stress in the renal tissues through the induction of the mitochondria uncoupling protein UCP2. GHSR-null mice exhibited renal dysfunction with an increase in oxidative stress, fibrosis and senescent changes as compared to WT littermates, indicating that endogenous Ghrelin/GHSR systems were essential for the regulation of ROS levels of the kidney. Our data provide compelling evidence for a novel strategy against the progression of renal insufficiency.

## Materials and Methods

### Ethics Statement

This study was performed in accordance with the institutional guidelines of the Animal Care and Experimentation Committee at Keio University. The experimental protocols were approved by the Animal Care and Experimentation Committee at Keio University (No. 09119). All surgery was performed under sodium pentobarbital anesthesia, and all efforts were made to minimize suffering. At the end of the experiments, the mice were euthanized by intraperitoneal injection of sodium pentobarbital.

### Animal experimental protocol

Sixteen-week-old male C57/BL6J mice (Nippon Clea, Tokyo, Japan) were divided into four groups (n = 8 per group). Three groups were treated with continuous AngII (1500 ng/kg/day, Peptide Institute Inc., Osaka, Japan) infusion during 28 days by using an Alzet micro-osmotic pump (Model 1004D; Durect Co., Cupertino, CA). The dose of 1500 ng/kg/min AngII is most commonly used in rodents and causes relevant elevations of blood pressure. One group received Ghrelin (100 µg/kg/day) by daily intraperitoneal injection for 14 days. One group was administered hydralazine orally at the dose of 25 mg/kg/day [14]. Blood pressure was measured weekly by tail-cuff plethysomography (TSE 209000; TSE Systems) as previously described [40]. Body weight and chow intake were monitored every week. Five days before sacrifice, animals were housed in metabolic cages over 24 hours for urine collection. Daily urinary excretion of protein, NAG (beta-N-acetylglucosaminidase) and neutrophil-associated lipocalin (NGAL) were measured by ELISA (Quantikine, R&D Systems, Minneapolis) and expressed as normalized by urinary excretion of creatinine.

### Histomorphology and Immunohistochemistry

Kidney tissues were fixed with PBS containing 4% paraformaldehyde and embedded in paraffin. Sections were cut 5 µm and used in immunohistochemical staining. Immunohistochemical staining was performed using primary antibody against 4-Hydroxynonenal-2-nonenal (4HNE) (NIKKEN SEIL Co.,Fukuroi, Japan) and GHSR (Sigma-Aldrich Co., St. Louis, MO). Horseradish peroxidase-conjugated anti-mouse and anti-rabbit IgG antibodies (Dako, Glostrup, Denmark) were used for secondary antibody.

### Electromicroscopic analysis

The samples were fixed with 2% paraformaldehyde (PFA), 2% glutaraldehyde (Distilled EM grade, Electron Microscopy Sciences, Hatfield, PA) in 0.1 M PBS pH 7.4 at 4°C overnight. After the fixation, the samples were rinsed three times with 0.1 M PBS for 30 minutes, followed by post-fixation with 2% osmium tetroxide(OsO_4_) in PBS at 4°C for two hours. The samples were then infiltrated with propylene oxide (PO) twice for 15 min and put them into a mixture of PO and resin (Quetol-812; Nisshin EM Co.,Tokyo, Japan) for one hour, followed by keeping the cap of tube open, and PO was volatilized overnight. The samples was transferred to a fresh 100% resin, and polymerized at 60°C for 48 hours. The blocks were ultra-thin sectioned at 70 nm with a diamond knife using a ultramicrotome (ULTRACUT UCT; Leica, Wetzlar, Germany) and sections were placed on copper grids. They were stained with 2% uranyl acetate at room temparature for 15 min. and then rinsed with distilled water followed by being secondary-stained with Lead stain solution (Sigma-Aldrich) at room temparature for three minutes. The grids were observed by a transmission electron microscope (JEM-1200EM; JEOL Ltd., Akishima, Japan) at an acceleration voltage of 80 kv. Digital images (2048×2048pixels) were taken with a CCD camera (VELETA; Olympus Soft Imaging Solutions GmbH, Münster, Germany).

### Cell culture and experimental protocols

The human renal proximal tubular cell line HK-2 was purchased from American Type Culture Collection (Rockville, MD, USA) and cultured in Dulbecco's Modified Eagle Medium Nutrient Mixture F-12 (DMEM/F12; GIBCO Life Technologies, Foster City, CA) with 10% bovine serum (FBS), penicillin and streptomycin in a humidified 5% CO_2_ incubator at 37°C. The cells were seeded at a density of 1×10^6^ cells per well. After HK-2 cells were grown to 80% confluence and made quiescent by serum starvation for 24 hours. Ghrelin at three different concentrations of 1, 10 and 100 nM, Des-acyl Ghrelin (Peptide Institute Inc., Osaka, Japan), inactive form of Ghrelin [1] at 10 nM and Irbesartan at 1 µM were added 30 minutes before the treatment with AngII at 1 µM (Sigma-Aldrich). Forty-eight hours after stimulation, cell lysates and culture supernatants were obtained. AMP-kinase inhibitor, Compound C was purchased from Calbiochem (San Diego, CA). The concentrations of TGF-β and PAI-1 were measured with the quantitative sandwich enzyme immunoassay technique (R&D Systems).

### Senescence-associated β-galactosidase staining

Renal tubular cells (HK-2 cell) and kidney tissues were washed with PBS and fixed in 4% paraformaldehyde in PBS for 30 minutes, followed by three washes with PBS. The degree of senescence in renal tubular cells and the kidney tissues were evaluated using the senescence-associated β-galactosidase (SA β-gal) staining kit (Sigma-Aldrich) according to manufacturer's instructions. The samples were incubated for 24 hours at 37°C β-gal staining solutions (pH 6.0) containing 1 mg/ml 5-bromo-4-chrolo-3-indlyl β-D-galavtopylanoside (X-gal), 5 mM potassium fericynide, 150 mM NaCl, 2 mM MgCl_2_, 0.01% Nonidet-40.

After the stained kidney tissues were photographed, the samples were immersed in OCT compounds (Miles Inc., Monrovia, CA) and snap-frozen in liquid nitrogen to prepare cryostat sections. The frozen sections (5 µm) were subjected to immunohistochemistry. Senescent renal tubular cells (stained blue) were observed with microscope and digitally photographed.

### UCP2 siRNA experiments

Small interfering RNAs (siRNAs) for UCP2 were prepared by Dharmacon (Lafayette, CO). The targeted sequences to silence the transcription of human UCP2 were 5′-GCAUCGGCCUGUAUGAUUC-3′. We used siGENOME Non-Targeting siRNA with at least four mismatches to any human, mouse or rat gene as the negative control siRNA. The siRNA molecules (final concentration at 25 nM) were transfected into HK-2 cells at 60% confluence using Lipofectamine 2000 (Invitrogen, Carlsbad,CA) according to manufacturer's instructions. Forty-eight hours after transfection, we administered Ghrelin 100 nM and incubated the cells for 24 h.

### Quantification of mitochondrial DNA copy number

Total DNA was extracted with the aid of a Qiamp DNA mini kit (Qiagen, Valencia, CA) from the HK-2 cells. The mitochondrial DNA copy number was determined by means of quantitative PCR analysis (ABI7500 Real-Time PCR System, Applied Biosystems, Foster City, CA), using specific primers for the mitochondrial DNA encoded COX 1 gene and the nuclear DNA encoded lipoprotein lipase (LPL) gene, as follows; the COX1 gene, 5′-TCG CCA TCA TAT TCG TAG GAG-3′ and 5′-GTA GCG TCG TGG TAT TCC TGA-3′; and the LPL gene,5′-CGAGTCGTCTTTCTCCT GATGAT-3′ and 5′-TTCTGGATTCCA ATGCTTCGA-3′ [42]. Samples were assayed in triplicate.

### Quantification of mitochondrial mass, ROS production and membrane potential

Mitochondrial mass, mitochondrial ROS (Mit ROS) production, and membrane potential of the HK-2 cells were determined with the aid of the fluorescent dyes MitoTracker Green FM and MitoSOX Red, which can selectively detect superoxide derived from mitochondria and Rhodamine 123 (Molecular Probes, Eugene, OR), respectively, with the same procedures as described elsewhere [43], [44]. To normalize the data, we used Hoechst 33342 (Molecular Probes, Wako, Osaka, Japan) for nuclear staining. The value for mitochondrial mass was normalized by that for nuclei, and the value for mitochondrial ROS and membrane potential was normalized by that for mitochondrial density. After the cells were cultured with the aforementioned agents for 24 hours, they were treated with the dyes for 10 min, and washed twice with warm PBS. The fluorescent intensity was measured with a Wallac ARVO SX multiplate reader (Perkin-Elmer, Norwalk, CT).

### Quantification of total cellular ROS and superoxide production

Total oxidative stress and superoxide derived from HK-2 cells were measured by total ROS/Superoxide Detection Kit (Enzo Life Science, Farmingdale, NY) as manufacturer's directions. This kit enables detection of comparative levels of total ROS and also allows determination of superoxide production using two specific fluorescent probes.

### GHSR-null mice

GHSR-null mice were kindly provided from Professor Jeffrey M. Zigman (The University of Texas Southwestern Medical Center). These mice were generated on C57BL/6 background as described previously [21]. We crossed GHSR null mice onto C57BL/6 mice (Clea Japan Inc., Tokyo, Japan) to obtain the F1 generation. We further crossed these F1 mice for two generations to get homozygous null mice for GHSR and wild type littermates. We used these mice of two genotypes for the experiment. Genomic DNA was isolated from tail biopsies at four weeks of age using a DNeasy kit (Qiagen Inc., Valencia, CA) and screening of genomic DNA samples was done by polymerase chain reaction using transgene-specific oligonucleotide primers, GHSR-null genotype primers; Primer M204: CGGTCTCCACCCTTCATTACTTTA and Primer M274: CAGATGTAGCTAAAAGGCCTATCACAAACT. WT genotype; Primer M204: CGGTCTCCACCCTTCATTACTTTA and Primer M313: GATGCTTGGGGAA GAGAGAAGTGA. These groups were treated with continuous AngII (500 ng/kg/day, Peptide Institute Inc., Osaka, Japan) infusion during 28 days by using an Alzet micro-osmotic pump (Model 1004D; Durect Co., Cupertino, CA).

### RNA extraction and real-time PCR fast SYBR green master mix

Total RNA was extracted from renal proximal tubular cells (HK-2) and the mouse kidney tissues using TRIzol reagent. Equal amounts (1 µg) of total RNA from each sample were converted to cDNA by PrimeScript RT reagent Kit with gDNA Eraser (TaKaRa, Ohtsu, Japan) in a 20 µl reaction volume. Real-time PCR was performed using an ABI Step One Plus sequence detector (PE Applied Biosystems, Tokyo, Japan).

Amplification products were analyzed by a melting curve, which confirmed the presence of a single PCR product in all reactions. Levels of mRNA were normalized to those of GAPDH. The primer sequences were as follows: TGF-β, sense 5′-GCACGTGGAGCTCTACCA-3′ and antisense 5′-CAGCCGGTTGCTGAGGTA -3′; PAI-1, sense5′-CTCTCTCTGCCCTCACCAAC-3′ and antisense 5′-GTGGAGAGG CTCTTGGTCTG-3′; GAPDH, sense 5′-GCACCGTCAAGGCTGAGAAC-3′and antisense 5′-TGGTGAAGACGCCAGTGGA-3′ for HK-2cells. TGF-β, sense 5′-GTGTGGAGCAACATGTGGAACTCTA-3′ and antisense 5′-TTGGTTCAG CCACTGCCGTA-3′; PAI-1, sense 5′-GAGTGGCCTGCTA GGAAATCCATTC-3′ and antisense 5′-GACCTTGCCAAGGTGATGCTTGGCAAC-3′; GAPDH, sense 5′-ATGTTCCAGTATGACTCCACTCACG-3′ and antisense 5′-GAAGACACCA GTAGACTCCACGACA -3′ for mouse. The amplification program was 95°C for three minutes, and then 40 cycles consisting of 95°C for 10 seconds, 62°C for 10 seconds, and 72°C for 10 seconds.

### Immunoblotting

Kidney tissues were removed at the sacrifice and snap frozen. Tissues were lysed and sonicated in lysis buffer and centrifuged at 15,000 *g* for 15 minutes. Supernatant aliquots were subject to immunoblotting using primary antibody against p53, p21 (Cell Signaling Technology, Frankfurt, Germany), and UCP2 (Calbiochem, Darmstadt, Germany). After blots were incubated with secondary antibody (HRP-linked whole body anti-rabbit or -mouse IgG, GE healthcare, Backhamshire, England), immunoreactive bands were detected using an ECL detection kit (Amersham Biosciences, Uppsala, Sweden).

### Statistical Analysis

Data are expressed as mean±SEM. One-way analysis of variance was used to determine significant differences among groups. In the overall analysis of variance, Kruskal-Wallis test for multiple comparisons was used to assess individual group differences. P<0.05 was considered statistically significant.

## Supporting Information

Figure S1 **Expression of GHSR and the effects of siRNA for UCP2 in HK-2 cells.** (A) Real-time PCR (left panel) and immunoblotting (right panel) revealed the expressions of GHSR in HK-2 cells. RT^−^ represents the results of real-time PCR without reverse transcription. Control; control cells, AngII; HK-2 cells treated with 1 µM of AngII, AngII+Ghr; HK-2 cells treated with AngII plus 10 nM of Ghrelin. AngII+Des-Ghr; HK-2 cells treated with AngII plus 10 nM of desacyl-Ghrelin. B, After the transfection of siRNA for UCP2, expressions of MnSOD (B), Zn/Cu SOD (C), UCP1 (D), and UCP3 (E) were examined by real-time PCR.
(TIF)

Figure S2 **Effects of Ghrelin on the phenotype of AngII-infused GHSR-null mice.** (A) Real-time PCR analysis using specific primers shows the mRNA expression of GHSR in the kidney. WT, wild type mice. GHSR^−/−^, GHSR-null mice. n = 6. (B–I) The effects of Ghrelin on the phenotype of GHSR^−/−^ mice infused with AngII. Fourteen-weeks treatment with Ghrelin did not affect blood pressure (B), serum levels of blood urea nitrogen (BUN, C) and creatinine (D) and urinary excretion of protein (E), neutrophil gelatinase-associated lipocalin (NGAL, F), and n-acetyl-galactasaminase (NAG, G). Urinary excretion of each marker was normalized by that of creatinine. (H) The expression of UCP2 was also unaffected by Ghrelin in AngII-infused GHSR-null mice. (I) Representative immunostaining for 4-Hydroxynonenal-2-nonenal (4HNE) of four experimental groups. Bar graph represents the quantification of immunostained areas. Scale bar; 50 µm. (B–I) **p<0.01 vs. GHSR^−/−^+NS, *p<0.05 vs. GHSR^−/−^+NS, n = 8.
(TIF)
