# Supplementary figures and images for: Ghrelin Protects against Renal Damages Induced by Angiotensin-II via an Antioxidative Stress Mechanism in Mice
Source: PLoS One. 2014 Apr 18;9(4):e94373. doi: 10.1371/journal.pone.0094373 (PMC3991592; doi:10.1371/journal.pone.0094373)

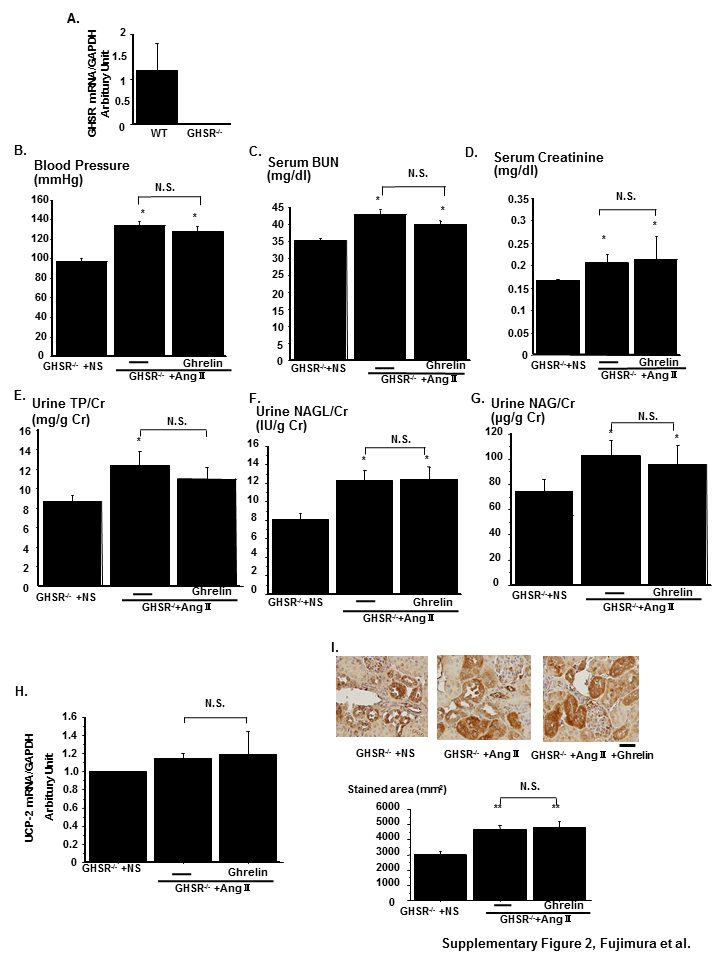

Supplement: Figure S2 — Effects of Ghrelin on the phenotype of AngII-infused GHSR-null mice. (A) Real-time PCR analysis using specific primers shows the mRNA expression of GHSR in the kidney. WT, wild type mice. GHSR−/−, GHSR-null mice. n = 6. (B–I) The effects of Ghrelin on the phenotype of GHSR−/− mice infused with AngII. Fourteen-weeks treatment with Ghrelin did not affect blood pressure (B), serum levels of blood urea nitrogen (BUN, C) and creatinine (D) and urinary excretion of protein (E), neutrophil gelatinase-associated lipocalin (NGAL, F), and n-acetyl-galactasaminase (NAG, G). Urinary excretion of each marker was normalized by that of creatinine. (H) The expression of UCP2 was also unaffected by Ghrelin in AngII-infused GHSR-null mice. (I) Representative immunostaining for 4-Hydroxynonenal-2-nonenal (4HNE) of four experimental groups. Bar graph represents the quantification of immunostained areas. Scale bar; 50 µm. (B–I) **p<0.01 vs. GHSR−/−+NS, *p<0.05 vs. GHSR−/−+NS, n = 8. (TIF) [file pone.0094373.s002.tif]
